# Differential localization and limited cytotoxic potential of duodenal CD8^+^ T cells

**DOI:** 10.1172/jci.insight.154195

**Published:** 2022-02-08

**Authors:** Leonard Mvaya, Trevor Khaba, Agness E. Lakudzala, Thandeka Nkosi, Ndaru Jambo, Innocent Kadwala, Anstead Kankwatira, Priyanka D. Patel, Melita A. Gordon, Tonney S. Nyirenda, Kondwani C. Jambo, Zaza M. Ndhlovu

**Affiliations:** 1 Malawi-Liverpool-Wellcome Trust Clinical Research Programme, Blantyre, Malawi.; 2 HIV Pathogenesis Programme, Doris Duke Medical Research Institute, University of KwaZulu-Natal, Durban, South Africa.; 3 Africa Health Research Institute, Durban, South Africa.; 4 Department of Pathology, Kamuzu University of Health Sciences, Blantyre, Malawi.; 5 Institute of Infection, Veterinary and Ecological Sciences, University of Liverpool, Liverpool, United Kingdom.; 6 Department of Clinical Sciences, Liverpool School of Tropical Medicine, Liverpool, United Kingdom.; 7 Ragon Institute of MGH, MIT, and Harvard, Cambridge, Massachusetts, USA.

**Keywords:** AIDS/HIV, Immunology, Cellular immune response, T cells

## Abstract

The duodenum is a major site of HIV persistence during suppressive antiretroviral therapy despite harboring abundant tissue-resident memory (Trm) CD8^+^ T cells. The role of duodenal Trm CD8^+^ T cells in viral control is still not well defined. We examined the spatial localization, phenotype, and function of CD8^+^ T cells in the human duodenal tissue from people living with HIV (PLHIV) and healthy controls. We found that Trm (CD69^+^CD103^hi^) cells were the predominant CD8^+^ T cell population in the duodenum. Immunofluorescence imaging of the duodenal tissue revealed that CD103^+^CD8^+^ T cells were localized in the intraepithelial region, while CD103^–^CD8^+^ T cells and CD4^+^ T cells were mostly localized in the lamina propria (LP). Furthermore, HIV-specific CD8^+^ T cells were enriched in the CD69^+^CD103^–/lo^ population. However, the duodenal HIV-specific CD8^+^ Trm cells rarely expressed canonical molecules for potent cytolytic function (perforin and granzyme B) but were more polyfunctional than those from peripheral blood. Taken together, our results show that duodenal CD8^+^ Trm cells possess limited perforin-mediated cytolytic potential and are spatially separated from HIV-susceptible LP CD4^+^ T cells. This could contribute to HIV persistence in the duodenum and provides critical information for the design of cure therapies.

## Graphical abstract

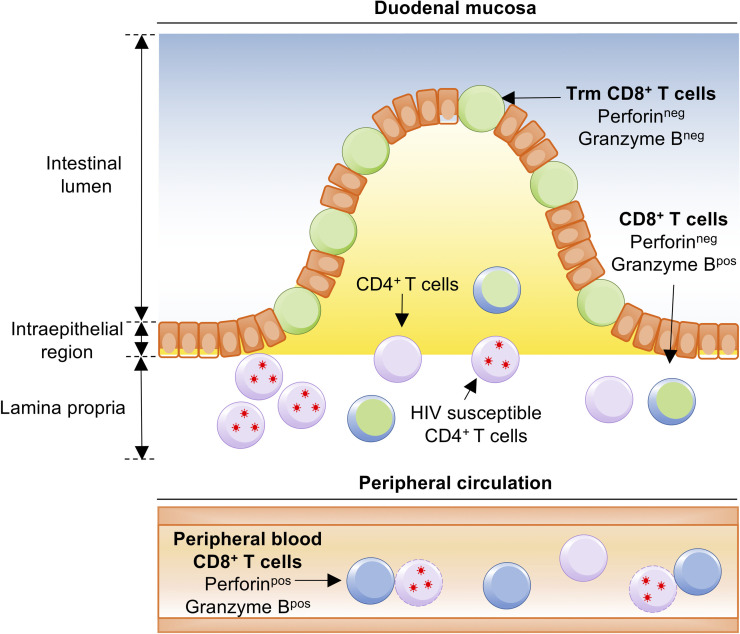

## Introduction

HIV remains a major global health challenge; however, the advent of antiretroviral therapy (ART) has significantly reduced HIV-related morbidity and mortality in both developed and developing countries (1). Although ART significantly reduces viral load to clinically undetectable levels in systemic circulation, it does not completely eliminate the virus (2–4). HIV establishes viral reservoirs in tissue and cellular reservoirs even after years of long-term suppressive ART, making it difficult to achieve a cure for HIV (5–8). As such, understanding HIV-specific immune responses within tissues is pivotal for the development of immune therapies capable of clearing the virus.

Multiple studies conducted using tissue samples from different organs have highlighted differences in immune responses within systemic circulation and tissue sites, indicating compartmentalization of immune responses (9–11). Lymph node and rectosigmoid mucosa resident CD8^+^ T cells have been shown to exhibit lower cytotoxicity than peripheral CD8^+^ T cells (12, 13). Within the tissue sites, resident immune cells occupy distinct spatial microenvironments (14, 15). Moreover, most of the research on resident immune cells has been conducted in animal models (16, 17). Thus, we do not yet fully understand the extent to which compartmentalized immune responses in human tissues contribute to HIV clearance or persistence, mainly because of the invasive procedures required to obtain appropriate tissue samples.

One of the major sites of HIV replication and persistence is the gut mucosa, which is thought to contain most of the lymphocytes in humans, making it a major site of immune responses (18). Therefore, it is not surprising that there is rapid depletion of mucosal CD4^+^ T cells in the early stages of HIV infection at this site (19). Fittingly, the gut has been proposed to be the largest reservoir of latently infected CD4^+^ T cells (18, 20). However, different regions of the gastrointestinal (GI) tract have specialized functions. There is, accordingly, heterogeneity in the architecture of the GI tract that influences the distribution of immune cells within the gut mucosa, which, in turn, results in different levels of HIV burden in different regions of the gut (21). For instance, it has been shown that HIV-associated disruption of immune cells differs between the ileal and rectal mucosa (21). Furthermore, there is differential ART-associated restoration of immune cell disruption between the small and large intestines (22).

The duodenum is a major site where HIV-associated immune cell disruption is markedly increased and is a major site of HIV persistence during suppressive ART (23, 24). Here, we investigated why the duodenum remains a major reservoir of HIV during suppressive ART despite harboring abundant resident CD8^+^ T cells. We examined the spatial localization and functional potential of bulk and HIV-specific CD8^+^ T cells in human duodenal tissue as potential contributors to poor viral control in the duodenum. By showing that tissue-resident memory CD8^+^ T cells are markedly less cytotoxic and are mostly spatially separated from HIV-susceptible lamina propria (LP) CD4^+^ T cells, this study provides critical information for the design of therapies that seek to eradicate HIV infection in the gut.

## Results

### Participant demographics.

We recruited 55 individuals comprising those classified as HIV uninfected (*n =* 17), untreated asymptomatic people living with HIV (PLHIV) (*n =* 22), and treated PLHIV (*n =* 16). A description of participant demographics, including sex, age, CD4 count, and viral load, are in Table 1. In summary, both the untreated and treated PLHIV had relatively high CD4 counts (median 552 vs. 413, *P =* 0.5691). The median duration on ART for the treated PLHIV was 5.5 years, and only 3 individuals had a detectable plasma viral load.

### HIV-associated disruption of duodenal T cell homeostasis is not restored with suppressive ART.

**Figure 1:**
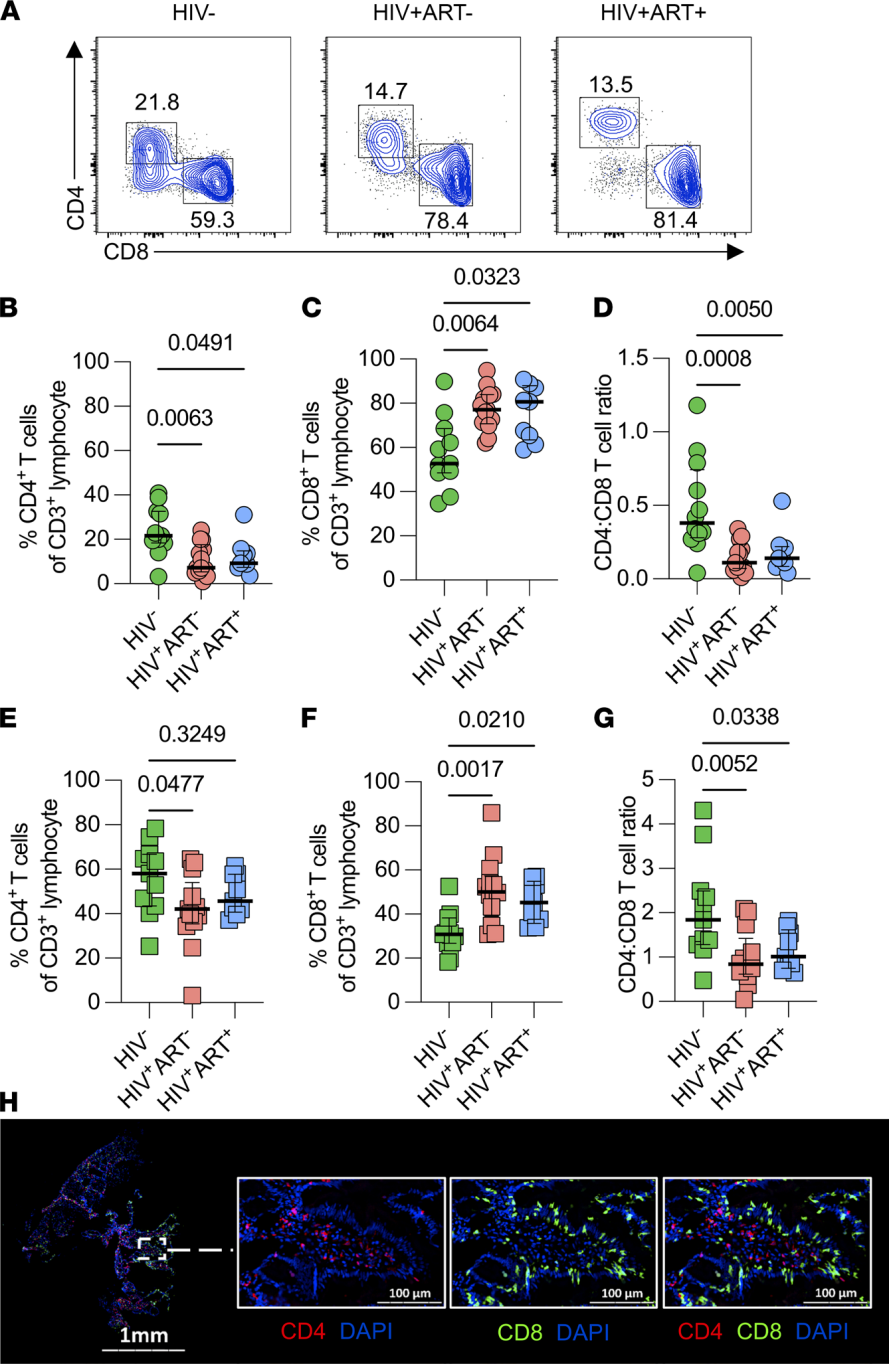
Identification of CD4^+^ and CD8^+^ T cells in the duodenal mucosa and peripheral circulation. DMNCs and PBMCs from HIV-uninfected adults and PLHIV were stained with fluorochrome-conjugated antibodies against surface markers of interest. (**A**) Representative flow cytometry plots showing CD4^+^ and CD8^+^ T cells in DMNC samples from healthy HIV-uninfected, ART-untreated and ART-treated adults. (**B**) Frequency of duodenal CD4^+^ T cells in healthy controls compared with ART-untreated and ART-treated. (**C**) Frequency of duodenal CD8^+^ T cells in healthy controls compared with ART-untreated and ART-treated. (**D**) Duodenal CD4^+^/CD8^+^ T cell ratio in healthy controls compared with ART-untreated and ART-treated PLHIV. (**E**) Frequency of peripheral blood CD4^+^ T cells in healthy controls compared with ART-untreated and ART-treated PLHIV. (**F**) Frequency of peripheral blood CD8^+^ T cells in healthy controls compared with ART-untreated and ART-treated PLHIV. (**G**) Peripheral blood CD4^+^/CD8^+^ T cell ratio in healthy controls compared with ART-untreated and ART-treated PLHIV. Cell proportions were assessed only in individuals with paired DMNC and PBMC samples (HIV−, *n =* 11; ART^–^, *n =* 13; ART^+^, *n =* 9) (**B**–**G**). Data were analyzed using Kruskal–Wallis test and adjusted for multiple comparisons (Dunn’s test) for different participant groups (**B**–**G**). (**H**) Representative IHC image of duodenal biopsy section showing CD4 (red), CD8 (green), and DAPI (blue).

The GI tract harbors the largest population of HIV-susceptible CD4^+^ T cells, which are rapidly and extensively depleted during acute HIV infection (19, 25). We investigated whether HIV-associated CD4^+^ T cell depletion in the duodenal mucosa is restored by ART. Paired duodenal tissue biopsies and peripheral blood samples were collected from individuals living with HIV (ART^–^ and ART^+^) and HIV-infected adults. Consistent with previous reports, flow cytometric analysis revealed lower proportions of CD4^+^ T cells in untreated individuals infected with HIV compared with healthy controls (*P =* 0.0063) and ART did not restore CD4^+^ T cells (Figure 1, A and B; Table 1; Supplemental Figure 1A for gating strategy; supplemental material available online with this article, https://doi.org/10.1172/jci.insight.154195DS1). CD4^+^ T cell depletion was associated with elevated proportions of CD8^+^ T cells (Figure 1C). Although the increase in the relative proportions of CD8^+^ T cells could be attributed to a proportional decrease in CD4^+^ T cells, there is evidence suggesting that acute HIV infection is associated with an accumulation of CD8^+^ T cells that traffic to the duodenum, which could contribute to increased CD8^+^ T cell proportions in the duodenal mucosa (23, 26). The duodenal CD4/CD8 T cell ratio was lower in both ART^–^ (*P =* 0.0010) and ART^+^ (*P =* 0.0057) PLHIV compared with HIV-infected adults (Figure 1D). Notably, in peripheral blood, the proportion of CD4^+^ T cells (*P =* 0.0477) was lower in ART^–^ PLHIV but not in ART^+^ PLHIV compared with HIV-infected adults (Figure 1E), confirming successful peripheral CD4^+^ T cell reconstitution by ART treatment. The proportion of CD8^+^ T cells and CD4/CD8 T cell ratio were higher in ART^–^ PLHIV and ART^+^ PLHIV compared with HIV-infected adults (Figure 1, F and G). Overall, the proportion of CD4^+^ T cells was lower and CD8^+^ T cells higher in the duodenum than blood, irrespective of HIV status (Supplemental Figure 1, B and C). We further evaluated the abundance of duodenal CD8^+^ T cells using FFPE duodenal tissues to detect CD4^+^ and CD8^+^ T cells by immunofluorescence microscopy (Figure 1H and Supplemental Figure 1D). Consistent with the flow cytometry data, quantitative image analysis of 7 duodenal biopsies from PLHIV and HIV-uninfected individuals revealed there was a greater density of CD8^+^ T cells than CD4^+^ T cells within the duodenum (*P =* 0.0156; Supplemental Figure 1E). Collectively, these findings support previous studies that have shown that ART does not fully restore T cell homeostasis in the duodenum (22, 24).

### Differential spatial localization of duodenal CD4^+^ and CD8^+^ T cells is consistent with CD103 expression.

**Figure 2:**
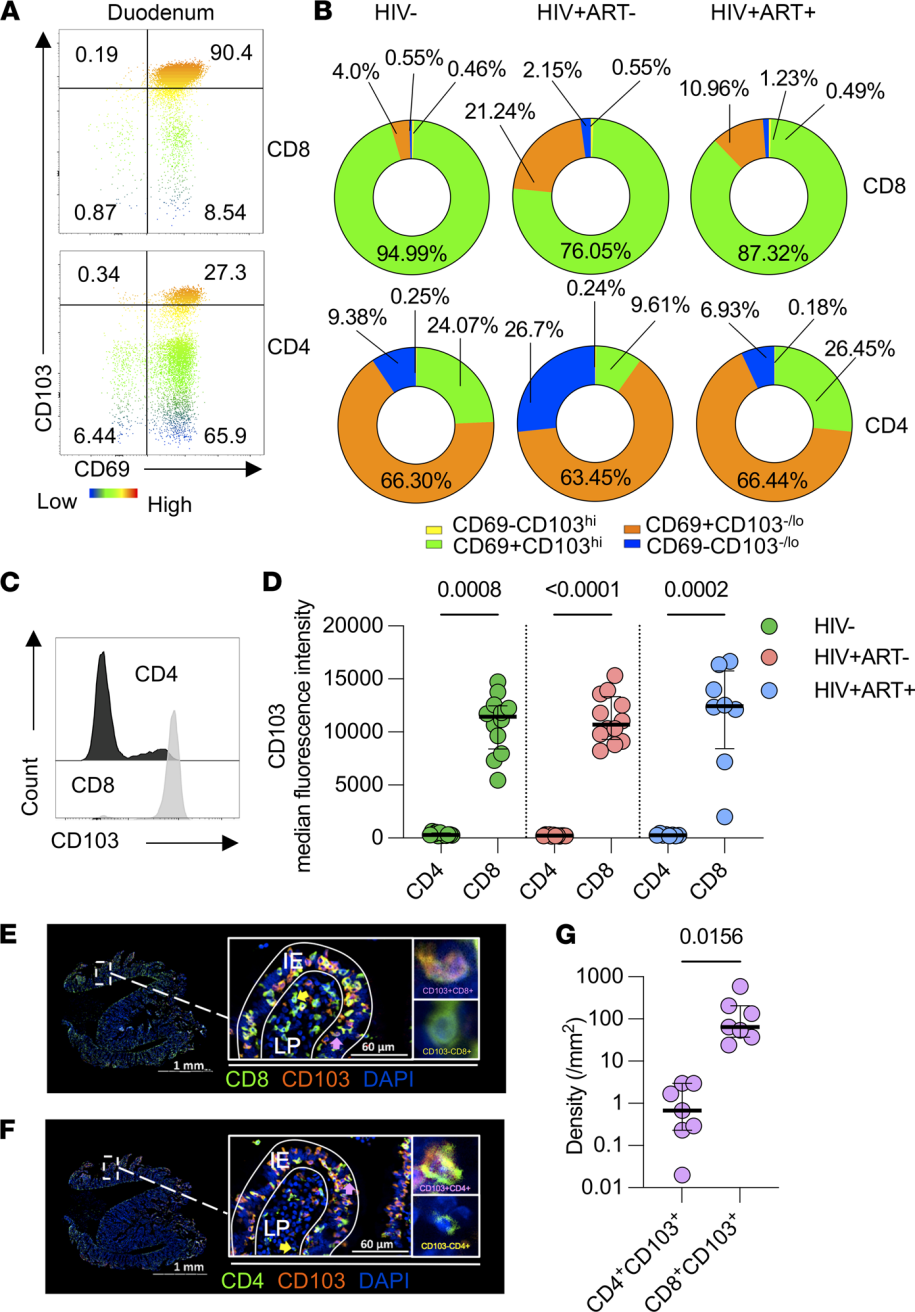
Characterization and spatial localization of resident memory CD4^+^ and CD8^+^ T cells in the duodenum. DMNCs from HIV-uninfected adults and PLHIV were stained with fluorochrome-conjugated antibodies against surface markers of interest. FFPE duodenal tissues were used to evaluate spatial localization of CD4^+^ and CD8^+^ T cells by immunofluorescence microscopy. (**A**) Representative flow cytometry plots showing identification of duodenal CD8^+^ and CD4^+^ T cells expressing different combinations of CD69 and CD103. (**B**) Pie charts representing the median proportion of duodenal CD8^+^ and CD4^+^ T cells expressing different combinations of CD69 and CD103 in different participant groups (HIV−, *n =* 13; ART^–^, *n =* 13; ART^+^, *n =* 10). (**C**) Representative histogram showing CD103 expression by duodenal CD4^+^ and CD8^+^ T cells. (**D**) CD103 expression intensity in duodenal CD4^+^ T cells compared with CD8^+^ T cells in different study participant groups (HIV−, *n =* 12; ART^–^, *n =* 13; ART^+^, *n =* 8). Data were analyzed using Kruskal–Wallis test and adjusted for multiple comparisons (2-stage Benjamini, Krieger, & Yekutieli) for different participant groups. (**E**) Representative IHC image of duodenal biopsy section showing CD8 (green), CD103 (orange), and DAPI (blue) staining within the delineated compartments defined as the IE region and the LP. (**F**) Representative IHC image of duodenal biopsy section showing CD4 (green), CD103 (orange), and DAPI (blue) staining within the delineated compartments defined as the IE region and the LP (**G**). (**H**) Density of CD103 expressing CD4^+^ and CD8^+^ T cells within duodenal tissue from PLHIV (ART^–^, *n =* 2; ART^+^, *n =* 2) and HIV-uninfected individuals (*n =* 3). Data were analyzed using Wilcoxon’s test (**G**).

Next, we investigated spatial localization of resident memory CD4^+^ and CD8^+^ T cells in the duodenum. CD69 and CD103 (αE integrin) expression are regarded as markers of tissue-resident memory (Trm) T cells (27). CD103 associates with β7 to form CD103/β7, an integrin which binds to the epithelial cell junctional protein, E-cadherin, allowing Trm cells to localize at the mucosal epithelium (28). In this report, we defined Trm CD8^+^ T cells as CD69^+^CD103^hi^ and identified further subpopulations defined as CD69^+^CD103^–/lo^ and CD69^–^CD103^–/lo^ (Figure 2A and Supplemental Figure 2A). CD103 high and negative/low populations were gated visually as previously described (29). Phenotypic analysis identified Trm CD8^+^ T cells (> 75%) and CD69^+^CD103^–/lo^ CD4^+^ T cells (> 60%) as the predominant populations in all study groups in the duodenum (Figure 2B), whereas CD69^–^CD103^–/lo^ expressing CD4^+^ and CD8^+^ T cells were the predominant subsets in blood (Supplemental Figure 2B). ART^–^ PLHIV had significantly higher proportions of duodenal CD69^+^CD103^–/lo^ CD8^+^ T cells and CD69^–^CD103^–/lo^ CD4^+^ T cells than HIV-infected adults (median percentage: 21.2% [IQR 8.7–35.4] vs. 4.0% [IQR 2.6–8.1]; median percentage: 26.3% [IQR 10.3–43.7] vs. 8.7% [IQR 4.0–16]) (Figure 2B). Further flow cytometric analysis of cell suspensions isolated from duodenal tissues of PLHIV (ART^–^, *n =* 12; ART^+^, *n =* 8) and HIV-infected adults (*n =* 12) revealed higher CD103 expression on CD8^+^ T cells than CD4^+^ T cells in duodenal tissue (Figure 2, C and D), suggesting duodenal CD8^+^ T cells have a greater propensity to adhere to the duodenal mucosal epithelium.

Having observed differences in CD69 and CD103 coexpression patterns between CD4^+^ and CD8^+^ T cells, we hypothesized that CD4^+^ and CD8^+^ T cells localize in different regions of the duodenal mucosa as has been observed in other sites of the GI tract (29). Immunofluorescence microscopy was used to evaluate spatial localization of CD4^+^ and CD8^+^ T. Imaging analysis revealed CD8^+^ T cells mostly colocalized with CD103 at the duodenal epithelium, while CD103^–^ CD8^+^ T cells were mostly localized in the LP (Figure 2E and Supplemental Figure 3). In contrast, CD4^+^ T cells were mostly CD103^–^ and resided in the LP, whereas CD103^+^ CD4^+^ T cells resided in the duodenal epithelium (Figure 2F and Supplemental Figure 3). Overall, consistent with flow cytometry analysis (Figure 2D), quantitative image analysis of 7 duodenal biopsies from PLHIV and HIV-uninfected individuals showed significantly greater density of CD103 expression on CD8^+^ T cells in duodenal tissue relative to CD4^+^ T cells (*P =* 0.0156; Figure 2G). Together, these data show that duodenal tissue harbors greater densities of tissue-resident CD8^+^ T cells compared with CD4^+^ T cells. We also show that Trm CD8^+^ T cells are mostly intraepithelial (IE) where they largely spatially segregated from an abundance of HIV-susceptible CD4^+^ T cell populations in the LP.

### Duodenal CD8^+^ T cells rarely coexpress cytolytic molecules and their expression of CD103 inversely correlates with granzyme B.

**Figure 3:**
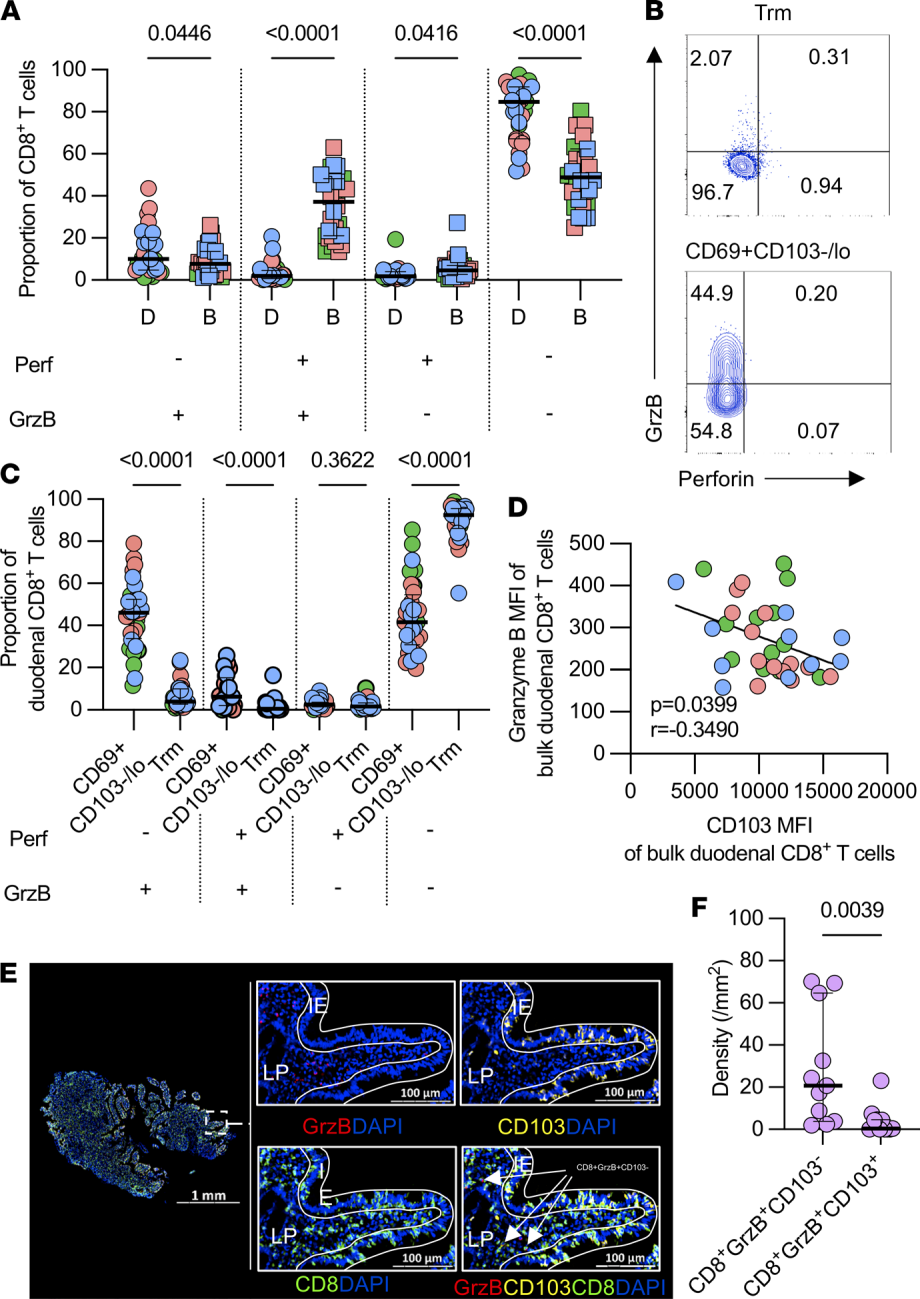
Characterization of cytotoxic potential of duodenal and peripheral blood CD8^+^ T cells. Unstimulated DMNCs and PBMCs from HIV-uninfected adults and PLHIV were stained with fluorochrome-conjugated antibodies against surface markers of interest. Intracellular staining was done to detect preformed perforin and granzyme B within CD8^+^ T cells. (**A**) Comparison of preformed perforin and granzyme B expression by duodenal and peripheral blood CD8^+^ T cells in a collated group of HIV-uninfected individuals and PLHIV (HIV−, *n =* 9; ART^–^, *n =* 13; ART^+^, *n =* 10). (**B**) Representative flow cytometry plots showing preformed perforin and granzyme B expression in unstimulated Trm and CD69^+^CD103^–/lo^ duodenal CD8^+^ T cells from a healthy control. (**C**) Comparison of preformed perforin and granzyme B expression by Trm and CD69^+^CD103^–/lo^ CD8^+^ T cells in a collated group of HIV-uninfected individuals and PLHIV (HIV−, *n =* 12; ART^–^, *n =* 13; ART^+^, *n =* 10). Data were analyzed using repeated measures 1-way ANOVA test (**A, C**). (**D**) Association between CD103 and granzyme B expression in bulk duodenal CD8^+^ T cells from all study participant groups (HIV−, *n =* 12; ART^–^, *n =* 13; ART^+^, *n =* 10). Data were analyzed using Pearson’s correlation test. (**E**) Representative IHC image of duodenal biopsy section showing granzyme B (red), CD103 (yellow), CD8 (green), and DAPI (blue) staining. (**F**) Density of duodenal mucosa granzyme B expressing CD8^+^ T cells, which are either CD103^+^ or CD103^–^. Data were analyzed using Mann–Whitney test.

Next, we assessed the cytolytic potential of duodenal CD8^+^ T cells, testing the hypothesis that spatial localization within duodenal tissue influence CD8^+^ T cell antiviral function. We measured the expression of preformed perforin and granzyme B, as surrogates of cytolytic potential (Gating strategy, Supplemental Figure 4, A and B). Overall, the frequency of CD8^+^ T cells coexpressing perforin and granzyme B were lower in the duodenum compared with blood, regardless of HIV status (median percentage: 2.1% [IQR 0.8–4.7] vs. 36.2% [IQR 21.1–47.9], *P <* 0.0001; Figure 3A). Though rare, CD69^+^CD103^–/lo^ CD8^+^ T cells had higher proportions of perforin^+^ granzyme B^+^ compared with Trm CD8^+^ T cells, irrespective of HIV status (median percentage: 4.1% [IQR 1.1–12.5] vs. 0.5% [IQR 0.1–1.2], *P <* 0.0001; Figure 3, B and C). Furthermore, we observed an inverse correlation between CD103^+^CD8^+^ T cells and granzyme B expression (Figure 3D). Quantitative image analysis showed greater density of CD103^–^GrzB^+^ CD8^+^ T cells than CD103^+^GrzB^+^ CD8^+^ T cells within the duodenal tissue (*P =* 0.0039; Figure 3, E and F; and Supplemental Figure 5). Together, these data show that duodenal CD8^+^ T cells have predominantly reduced cytolytic potential compared with peripheral blood CD8^+^ T cells. However, the CD69^+^CD103^–/lo^ CD8^+^ T cells possess a higher propensity for harboring preformed granzyme B than the Trm CD8^+^ T cells.

### HIV-specific CD8^+^ T cells are widely distributed within the duodenal tissue and exhibit reduced cytolytic potential.

**Figure 4:**
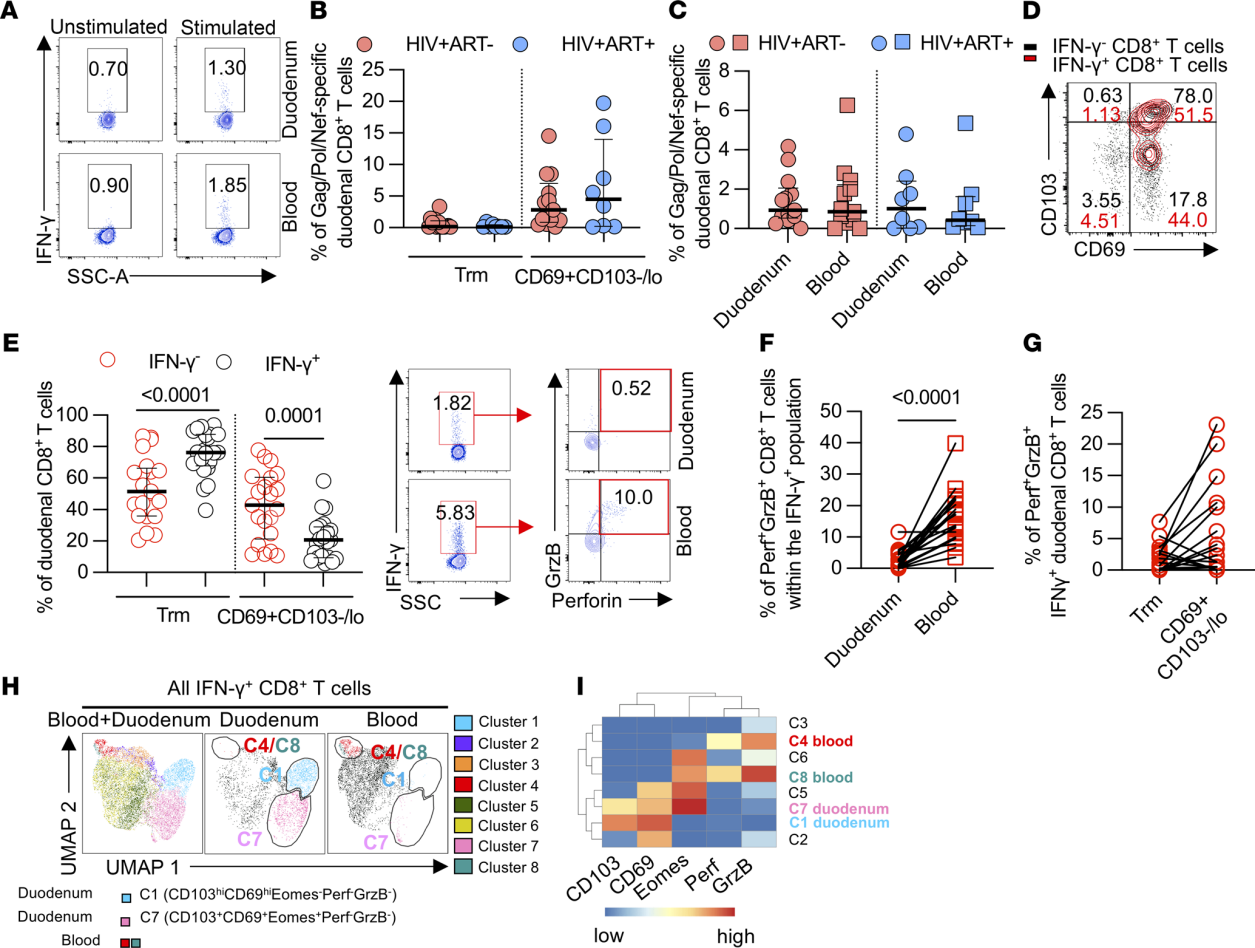
Characterization of the phenotype and cytotoxic potential of HIV-specific CD8^+^ T cells from the duodenal mucosa and peripheral blood (PB). (**A**) Representative flow cytometry plots showing detection of duodenal and PB IFN-γ–producing CD8^+^ T cells following stimulation with pooled HIV peptides. (**B**) Comparison of Gag/Pol/Nef CD8^+^ T cell response between the duodenum and blood. (**C**) Comparison of Gag/Pol/Nef response between Trm and CD69^+^CD103^–/lo^ duodenal CD8^+^ T cells. Data were analyzed using Kruskal–Wallis test and adjusted for multiple comparisons (Dunn’s test) for different participant groups (ART^–^, *n =* 13; ART^+^, *n =* 8) (**B** and **C**). (**D**) Representative flow plot and comparison of the proportions of IFN-γ^–^ and IFN-γ^+^ duodenal CD8^+^ T cells in the Trm and CD69^+^CD103^–/lo^ populations (ART^–^, *n =* 13; ART^+^, *n =* 8). Data were analyzed using Wilcoxon’s test. (**E**) Representative flow cytometry plots showing detection of duodenal and PB IFN-γ^+^ CD8^+^ T cells expressing perforin and granzyme B. (**F**) Perforin and granzyme B coexpression in duodenal HIV-specific CD8^+^ T cells compared with that of PB HIV-specific CD8^+^ T cells. (**G**) Frequency of perforin and granzyme B coexpressing Trm and CD69^+^CD103^–/lo^ HIV-specific CD8^+^ T cells. Data were analyzed using Wilcoxon’s test (ART^–^, *n =* 13; ART^+^, *n =* 8) (**F-G**). (**H**) UMAP of duodenal and PB HIV-specific CD8^+^ T cells from PLHIV (ART^–^, *n =* 5; ART^+^, *n =* 5). Colors represent phenotypically distinct clusters detected by FlowSOM. (**I**) Heatmap illustrating HIV-specific CD8^+^ T cell median expression intensity of CD103, CD69, Eomes, Perforin, and granzyme B (columns) by each cluster (rows) detected by FlowSOM.

Having determined the phenotype, cytotoxicity potential, and topological distribution of bulk duodenal CD8^+^ T cells, we next measured the frequency and function of HIV-specific CD8^+^ T cells in the duodenum. We chose to stimulate duodenal CD8^+^ T cells with HIV Gag, Nef, and Pol overlapping peptide pools (OLPs) because multiple reports have shown HIV-specific CD8^+^ T cell responses are predominantly directed toward these HIV proteins (30–32). Using a 6-hour ex vivo stimulation assay, duodenal cells were stimulated with pooled HIV Gag, Pol, and Nef peptides followed by intracellular staining for effector molecules. Flow cytometry was used to measure HIV-specific duodenal and blood CD8^+^ T cells which were defined as IFN-γ–producing CD8^+^ T cells (Gating strategy, Supplemental Figure 6A). HIV-specific CD8^+^ T cells were readily detectable in the duodenum in both ART^–^ and ART^+^ PLHIV at frequencies similar to those in blood and the magnitude of the HIV-specific responses did not differ between ART^–^ and ART^+^ PLHIV in both compartments (Figure 4, A and B). Within the duodenum, the frequency of HIV-specific Trm or CD69^+^CD103^–/lo^ CD8^+^ T cells was also not different between ART^–^ and ART^+^ PLHIV (Figure 4C). In contrast to HIV-nonspecific IFN-γ^–^ duodenal CD8^+^ T cells, a significantly greater proportion of HIV-specific CD8^+^ T cells had a CD69^+^CD103^–/lo^ phenotype (*P <* 0.0001), whereas a lesser proportion of HIV-specific CD8^+^ T cells were Trm, irrespective of treatment status (Figure 4D). Coexpression of perforin and granzyme B was significantly lower in the duodenal HIV-specific CD8^+^ T cell population compared with those from blood (*P <* 0.001; Figure 4, E and F). The proportions of perforin^+^ granzyme B^+^ HIV-specific CD8^+^ T cells within the CD69^+^CD103^–/lo^ and TrmCD8^+^ T cell populations did not differ (*P =* 0.0505; Figure 4G). Furthermore, we did not observe an enrichment in perforin^+^ granzyme B^+^ cells in the duodenal HIV-specific response relative to the bulk duodenal CD8^+^ T cell populations (Supplemental Figure 6B).

To gain more nuanced insight into the phenotype, residence, and cytotoxicity potential of HIV-specific CD8^+^ T cells in the duodenum relative to blood, we further analyzed our flow data using Uniform Manifold Approximation and Projection (UMAP) and FlowSOM unsupervised dimensional reduction algorithms. The analysis assessed the expression of CD69, CD103, perforin, granzyme B, and eomesodermin (Eomes) by HIV-specific CD8^+^ T cells. Eomes is known to modulate CD8^+^ T cell cytotoxicity (33) and has been shown to positively correlate with granzyme B expression in rectal mucosa CD8^+^ T cells^34^. As such, Eomes was added to evaluate its association with duodenal HIV-specific CD8^+^ T cell cytotoxic potential. UMAP was applied to visualize the distribution of all HIV-specific CD8^+^ T cell populations from all compartments in a 2D space and FlowSOM was applied to map out phenotypically related HIV-specific CD8^+^ T cell clusters. The analysis identified 8 distinct HIV-specific CD8^+^ T cell clusters (Figure 4H). Cluster 1 (35.6%) and cluster 7 (25.4%) were unique to the duodenum and showed high coexpression of CD69 and CD103, but low expression of perforin and granzyme B. Interestingly, despite cluster 7 expressing similar markers to cluster 1, it uniquely expressed high levels of Eomes. Inconsistent with observations from the rectal mucosa (34), this Eomes-expressing cluster did not possess high levels of preformed granzyme B, highlighting potential intestinal site-specific differences in CD8^+^ T cell phenotypes and functions. Cluster 4 (8.44%) and cluster 8 (2.33%) were unique to blood and showed high coexpression of perforin and granzyme B, but low expression of CD69 and CD103. Consistent with the supervised analysis, the unsupervised analyses demonstrated that duodenal HIV-specific CD8^+^ T cells were less likely to harbor preformed perforin and granzyme B, which are canonical molecules for potent cytolytic function (Figure 4I).

### HIV-specific CD8^+^ T cells are more polyfunctional in the duodenum compared with peripheral blood.

**Figure 5:**
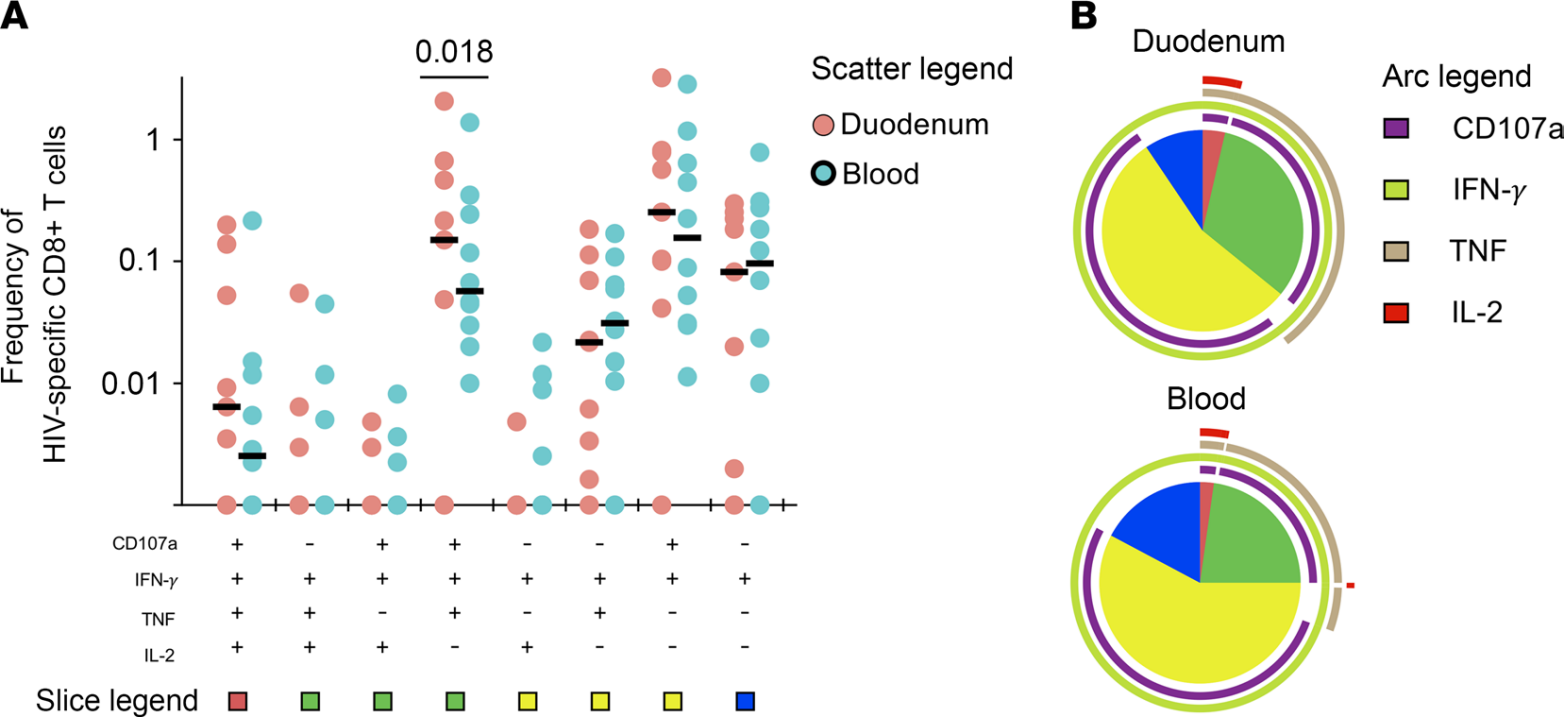
Functional profile of IFN-γ–producing duodenal and peripheral CD8^+^ T cells. DMNCs and PBMCs from PLHIV were stimulated with pooled HIV Gag, Pol, and Nef peptides for 6 hours and responses were measured by intracellular cytokine staining for TNF, IFN-γ, IL-2, and CD107a. The response was obtained by gating on singlets, lymphocytes, viable (LIVE/DEAD Aqua), CD3^+^ cells, CD8^+^ cells, IFN-γ^+^ cells, and combination of 3 cytokines and CD107a (ART^–^, *n =* 3; ART^+^, *n =* 6). (**A**) Dot plot represents the frequency of combinations of cytokines produced and/or degranulation response by duodenal and peripheral blood HIV-specific CD8^+^ T cells. Wilcoxon’s test was done among the dot plots using SPICE software. (**B**) Pie chart represents the mean distribution across subjects of monofunctional, bifunctional, and polyfunctional cytokine producing and/or degranulating duodenal and peripheral blood HIV-specific CD8^+^ T cells (color coded as shown). Size of each pie segment relates to the frequency of a monofunctional, bifunctional, and polyfunctional response. Arcs around the pie chart represent the particular cytokine produced and/or degranulation capacity of each particular response below arcs.

Having observed the poor cytolytic potential of the duodenal IFN-γ–producing HIV-specific CD8^+^ T cells, we next investigated whether duodenal CD8^+^ T cells employ other effector mechanisms for viral control. Nonperforin-mediated functions, including CD8^+^ T cell polyfunctionality defined by production of multiple cytokines, have been shown to correlate with viral control (35). We therefore measured the polyfunctional capacity of HIV-specific duodenal and blood CD8^+^ T cells in 6 ART-treated and 3 untreated participants who were selected based on sample availability. We defined polyfunctionality as the capacity of cells to simultaneously produce IFN-γ, TNF, IL-2, and/or degranulate (CD107a). Boolean gates on all cytokine secreting cells were used to enumerate polyfunctional HIV-1 specific CD8^+^ T cells. Our data show the frequency of CD107a^+^IFN-γ^+^TNF^+^IL-2^–^ CD8^+^ T cells was higher in the duodenum compared with blood (*P =* 0.018; Figure 5A). Furthermore, there was a trend toward more polyfunctional HIV-specific CD8^+^ T cells in the duodenum compared with peripheral blood cells (Figure 5B). These data suggest that duodenal CD8^+^ T cells could mediate their effector functions mainly through elaboration of proinflammatory cytokines.

### Relationship between duodenal HIV-specific CD8^+^ T cells and markers of HIV disease progression.

**Figure 6:**
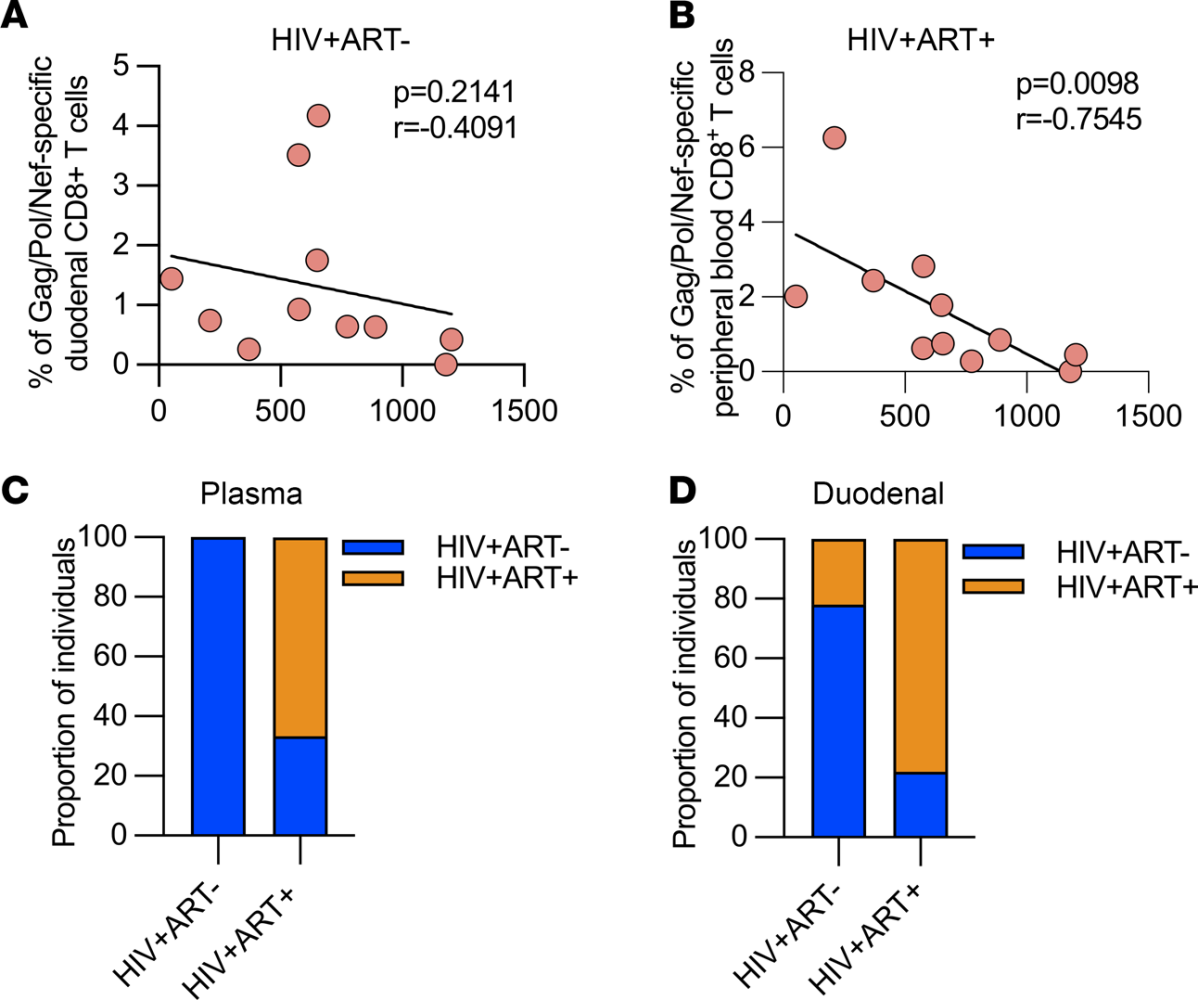
Evaluation of the relationship between duodenal HIV-specific CD8^+^ T cells and clinical indicators of HIV disease progression. (**A**) Association between frequency of duodenal HIV-specific CD8^+^ T cells and peripheral blood CD4^+^ T cell count (ART^–^, *n =* 11). (**B**) association between frequency of peripheral HIV-specific CD8^+^ T cells and peripheral blood CD4^+^ T cell count (ART^–^, *n =* 11). Data were analyzed using Pearson’s correlation test (**A** and **B**). (**C**) Frequency of individuals with detectable and undetectable plasma viral load (ART^–^, *n =* 9; ART^+^, *n =* 9) (**D**) Frequency of individuals with detectable and undetectable duodenal viral load (ART^–^, *n =* 9; ART^+^, *n =* 9).

Finally, we examined the relationship between duodenal HIV-specific CD8^+^ T cells and clinical indicators of HIV disease progression. We did not observe a correlation between duodenal HIV-specific CD8^+^ T cells with CD4^+^ T cell count (Figure 6A), but there was an inverse correlation between peripheral blood HIV-specific CD8^+^ T cells with CD4^+^ T cell count (*P =* 0.0098, *r* = 7,545; Figure 6B). We next explored the impact of ART on the viral burden in the duodenal tissue in a subset of individuals in whom we had paired HIV viral load data in plasma and duodenal tissue supernatant. We measured HIV RNA in duodenal tissue supernatant and plasma by quantitative PCR. As expected, the proportion of individuals with detectable plasma viral load was higher in untreated individuals than in treated individuals living with HIV (100% [9/9] vs. 33% [3/9]; *P =* 0.0090; Figure 6C). In agreement, a higher proportion of untreated PLHIV had detectable HIV RNA in duodenal lining fluid than PLHIV on ART (78% [7/9] vs. 22% [2/9]; *P =* 0.0567; Figure 6D). Moreover, there was a strong concordance (83% [15/18]) between the detection of HIV RNA between duodenal lining fluid and plasma. Collectively, these findings indicate that ART does effectively suppress viral production in the duodenal tissue.

## Discussion

The duodenal mucosa is one of the major sites of HIV-1 infection even during suppressive ART, despite harboring abundant tissue-resident CD8^+^ T cells. Our study investigated the spatial localization and functional potential of bulk and HIV-specific CD8^+^ T cells in human duodenal tissue, as potential contributors to poor viral control in the duodenum. Here, we show that Trm CD8^+^ T cells exhibited differential tissue localization potential relative to CD4^+^ T cells, suggesting spatial segregation from HIV-susceptible LP CD4^+^ T cell populations. Moreover, duodenal tissue-resident HIV-specific CD8^+^ T cells rarely coexpressed cytolytic molecules but have the potential to exert other effector functions including degranulation and cytokine production (IFN-γ and TNF-). Collectively, considering that cytolytic function is critical in the elimination of HIV-infected cells, our data suggest that low cytolytic potential of duodenal Trm CD8^+^ T cells and spatial segregation from HIV-susceptible LP CD4^+^ T cells could contribute to the persistence of latently HIV-infected CD4^+^ T cells in the gut mucosa.

Understanding the immunological and virologic features of the GI tract tissue that contributes to HIV persistence is critical to the development of vaccines and cure strategies. Moreover, whether HIV-specific GI tract tissue-resident CD8^+^ T cells play a significant role in HIV suppression remains a significant knowledge gap. Tissue-resident T cells are commonly defined by coexpression of CD69 and CD103 (9). CD69 prevents tissue egress of Trm T cells, while CD103 facilitates localization of Trm T cells in the IE region of the mucosa (28). This study identified 2 contributing factors that may impede HIV elimination in the duodenal mucosa. First, we showed that Trm CD8^+^ T cells have differential potential to localize in distinct duodenal tissue sites relative to HIV-susceptible CD4^+^ T cells. Second, we showed that Trm CD8^+^ T cells exhibit poor cytolytic potential compared with peripheral CD8^+^ T cells. Poor cytolytic function and segregation of HIV-specific CD8^+^ T cells from infected CD4^+^ T cells in the lymph nodes are associated with persistence of HIV in these sites (13, 36). In our study, differences in tissue localization potential between CD4^+^ and CD8^+^ T cells in duodenal tissue was revealed by fluorescent imaging and corroborated by flow cytometry-based immunophenotyping. Our analysis found CD103^+^CD8^+^ T cells exhibited poor cytolytic potential and exhibited a greater propensity to reside in the IE region, whereas CD4^+^ T cells mostly exhibited a phenotype associated with localization in the LP. The anatomical segregation between Trm CD8^+^ T cells and HIV-infected CD4^+^ T cells essentially makes the LP a potential haven for the virus to continually replicate unencumbered by Trm CD8^+^ T cells.

Effector functions of tissue-resident and recirculating HIV-specific CD8^+^ T cells in the host response against HIV in the GI tract tissue remain underexplored in humans. Previous studies have detected Trm CD8^+^ T cells in the rectal mucosa whose capacity to mount HIV-specific responses was particularly pronounced in elite controllers, while other studies have shown that elite controllers have high proportions of lymph node resident HIV-specific CD8^+^ T cells (37), suggesting that Trm CD8^+^ T cells likely contribute to HIV control. However, our data showed high proportions of Trm CD8^+^ T cells in the duodenal mucosa irrespective of HIV status, suggesting that HIV infection does not significantly perturb duodenal Trm CD8^+^ T cell distribution. Although these findings are inconsistent with reports from elite controllers, the observed differences may relate to differential distribution of Trm CD8^+^ T cells within the different sites of the GI tract (21). Prior studies have demonstrated that Trm CD8^+^ T cells in the rectosigmoid mucosa exhibit reduced coexpression of perforin and granzyme B compared with peripheral CD8^+^ T cells (12, 34). Concordantly, we showed that regardless of tissue localization, duodenal CD8^+^ T cells exhibited low perforin and granzyme B coexpression irrespective of HIV status. Further analysis of Trm CD8^+^ T cell cytotoxic capacity demonstrated an inverse correlation between duodenal CD8^+^ T cell CD103 and granzyme B expression. Reduced CD8^+^ T cell cytotoxic capacity in the gut mucosa may represent stringent regulation of perforin-mediated cytotoxicity to limit potential immunopathology that could disrupt the mucosal barrier (38). Considering that there is variability in abundance of HIV RNA and cell-associated DNA across different sites of the gut, it is possible that reduced CD8^+^ T cell cytotoxicity may not be uniform across the GI tract (18, 39). Thus, similar studies in multiple anatomical sites of GI tract tissue are warranted.

In line with previous studies, our data suggest that TrmCD8^+^ T cells could mediate effector functions mainly by cytokine release rather than cytotoxicity, which makes physiological sense given their limited migratory capacity (25, 40, 41). Our results are consistent with several reports that show that CD103 expression is associated with differential effector function of CD8^+^ T cells, including cytotoxicity and the capacity to produce multiple cytokines (42–44). Furthermore, there is evidence that suggests the down- or upregulation of Eomes by Trm CD8^+^ T cell is associated with differential cytokine production (45, 46). Indeed, we detected HIV-specific Trm CD8^+^ T cells exhibiting either high or low Eomes expression coupled with poor cytolytic molecule expression. These populations could represent Trm HIV-specific CD8^+^ T cell subpopulations that utilize other effector functions, including production of different functions, and warrant further investigation. Future studies should assess the relative contribution of nonperforin-mediated cytolytic effector functions, including production of HIV-suppressive factors such as RANTES, MIP-1 alpha, and MIP-1 beta (47), on control of HIV infection in the gut mucosa.

The main limitations of this study were that we only sampled from the duodenum. Sampling from multiple sites of the GI tract and longitudinal sampling would provide a more comprehensive picture of immune responses in this important anatomical site. However, this would be highly invasive. We also studied a relatively small sample size due to the invasive nature of the duodenal biopsy procedure and a more stringent protocol that leaned toward the safety and well-being of our study participants, since samples were taken for research purposes rather than for clinical indications. The small biopsy taken yielded a limited number of cells that precluded us from performing direct killing assays. We were also underpowered for evaluation of CD8^+^ T cell polyfunctionality between ART-treated and untreated PLHIV. Additional functional assays could have allowed a definitive assessment of the relative contribution of perforin-mediated cytolytic function and other noncytolytic effector functions of duodenal Trm CD8^+^ T cells in response to HIV infection. Finally, our study identified Trm CD8^+^ T cells through detection of the coexpression of CD69 and CD103; however, it is important to note that CD8^+^ T cell expression of CD69 and CD103 varies depending on the tissue being analyzed (48–50), bringing into question whether both markers can be used to definitively identify Trm CD8^+^ T cells.

Overall, our study demonstrates that Trm CD8^+^ T cells occupy unique niches in the duodenal tissue that are associated with differential, but limited, cytolytic potential. This could impact the ability of Trm CD8^+^ T cells in the duodenum to eliminate target HIV-infected CD4^+^ T cells, which primarily spatially localize in sites away from Trm CD8^+^ T cells. Collectively, these findings support the notion that future possible interventions for viral suppression or eradication in the gut mucosa might require measures that are independent of CD8^+^ T cell perforin-mediated mechanisms or that should focus on potentially relocating fully cytolytic CD8^+^ T cells to the site of HIV persistence.

## Methods

### Study participants.

We recruited healthy HIV-uninfected adults and asymptomatic PLHIV who were either on ongoing therapy or ART-naive. Participants were recruited from the HIV Voluntary Counselling and Testing (VCT) clinic at Queen Elizabeth Central Hospital (QECH) and Zingwangwa Health Centre in Blantyre, Malawi. All study participants were adults aged 18 to 60 with no clinical evidence of active disease and were willing to undergo upper GI tract endoscopy for research purposes. Exclusion criteria were febrile illness within 14 days preceding the screening appointment, antibiotic treatment within 14 days preceding the screening appointment, history of significant chronic disease (e.g., hypertension, diabetes mellitus) that could interfere with study completion or conduct as judged by the clinical team, any physiological contradiction to elective upper GI endoscopy (e.g., history of esophageal perforation, recent myocardial infarction, anticoagulation, pharyngeal diverticulum, head and neck surgery, or platelet count < 75 × 10^9^/mL), or confirmed pregnancy. ART-naive individuals were referred back to the VCT clinic to commence ART soon after undergoing endoscopy (within 36 hours following HIV diagnosis) in line with the Malawi national guidelines of “test and treat” strategy.

### Sample collection and experimental procedures.

An upper GI endoscopy was performed on all participants. A gastroscope was passed through the mouth into the participants’ esophagus, through the stomach, and into the duodenum. Flexible mucosal biopsy forceps were passed through a channel in the gastroscope with up to 15 × 3 mm punch biopsy samples taken from the lining of the small bowel. Two biopsy samples were fixed for IHC by placing them in a 15 mL Falcon tube containing paraformaldehyde. The remaining biopsy samples were placed in a 50 mL Falcon tube containing 20 mL of R-10 (Lonza Bioscience, RPMI-1640) with antibiotic/antimycotic (Sigma), heat inactivated FCS (10%), l-glutamine, nonessential amino acids, HEPES buffer solution and sodium pyruvate (all from Gibco). Paired peripheral blood samples were collected from study participants for CD4^+^ T cell count and peripheral blood mononuclear cell (PBMC) isolation.

### Sample processing.

Duodenal mononuclear cells (DMNCs) were isolated by first placing biopsy samples in a 50 mL falcon tube containing R10 media and carrying out enzymatic digestion by adding collagenase (Worthington/CLSS). Biopsies were mechanically agitated by placing them in a shaking incubator at 37°C for 30 minutes after which the biopsies were disrupted by repeatedly passing them through a blunt end needle. The disrupted tissue suspension was passed through a sterile 70 μm cell strainer and the digested cell suspension was collected. The collected tissue within the strainer was placed into a new falcon tube and the enzymatic digestion and tissue disruption process was repeated, after which a second cell suspension was collected. The collected DMNC suspensions were pooled together in R-10 and rested overnight at 37°C, 5% CO_2._ PBMCs were isolated from whole blood samples using density centrifugation, were then resuspended in R-10, and rested overnight at 37°C, 5% CO_2._ The counting of DMNCs and PBMCs isolated from each sample was performed the following day using a hemocytometer. Due to a limitation in cell numbers, we did not perform all experiments on all samples, as we were only able to isolate about 5 million cells per individual from the duodenal biopsies.

### IHC.

Formalin-fixed tissue sections were prepared into paraffin-embedded tissue blocks. These tissue blocks were cut into 4 μm sections and fixed onto Surgipath X-tra adhesive precleaned micro slides (Leica Biosystems) in preparation for antibody staining. The Opal polymer staining technology (Perkin Elmer) was utilized. Prepared slides were baked overnight to soften the paraffin wax encasing the tissue section. These were then deparaffinized in 2 vats of xylene (Sisco Research Laboratories) for 5 minutes each to expose the tissue. The tissue was then slowly rehydrated by submerging it in 100% ethanol for 2 minutes, 95% ethanol for 2 minutes, and 70% ethanol for 1 minute. Once rehydrated, the tissue was then denatured in boiling 1 × EnVision FLEX TRS High pH solution (Dako Denmark A/S) for 20 minutes to expose protein epitopes and then have its endogenous peroxidases blocked with REAL Peroxidase-Blocking Solution (Dako Denmark A/S) for 10 minutes followed by Bloxall Blocking solution (Vector Laboratories) for 10 minutes. The unconjugated primary antibody was added, followed by an OPAL polymer HRP Ms (Perkin Elmer) plus Rb secondary antibody for 20 minutes. For Brightfield, colorimetric staining occurred after incubation with prepared DAB substrate solution (Dako Denmark A/S) for 10 minutes, followed by counterstaining with Hematoxylin (Clinical Science Diagnostics) for 1 minute. Fluorescence staining occurred after incubation with a TRS Opal polymer (Perkin Elmer) for 10 minutes, followed by counterstaining with prepared DAPI (Perkin Elmer) solution for 5 minutes. For multiplexing with further antibodies, the tissue was boiled in AR6 buffer (Perkin Elmer) for 20 minutes to remove the previous antibody complex but leaving intact the covalently bound fluorescence marker; the steps were then repeated.

Antibodies used were optimized using single-color brightfield staining to determine their staining patterns. Signals were considered true when their staining patterns were consistent with the brightfield and their fluorescence intensities were significantly above background, allowing for the background threshold or lower threshold to be cut. The costaining of CD8 and CD4 showed no colocalization, which acts as a control to ensure that the AR6 stripping is successful.

### Quantitative image analysis.

Images were acquired on the Zeiss Axio Observer Inverted Microscope (TissueGenostics) running the TissueFAXS 6.129 software (TissueGenostics) with a 1 × optical zoom using an EC P|nN 40 × / 0.75 DICII objective on the channels DAPI, FITC, Texas Red, and Cy5. Analysis was performed using the TissueQuest 6.0.1.0136 software (TissueGenostics), where the channel markers were segmented based on the DAPI nuclei marker and the mean intensities set across for quantification. Densities and counts were generated and exported to excel. Delineation of the LP and IE regions was carried out morphologically similar to previously described methods (29). In brief, the DAPI signal intensity was adjusted and regions consisting of tightly packed nuclei in a linear conformation were identified as IE regions. Regions below the IE in which the DAPI signal was used to identify sparsely distributed cells were denoted as the LP.

### Flow cytometry assays.

The following fluorochrome-labeled monoclonal antibodies were used for staining in the surface immunophenotyping assay and the intracellular cytokine staining assay: CD3 (UCHT1: PerCP-Cy5.5, Bv785); CD4 (SK3: Bv605); CD8 (SK1: PerCP-Cy5.5, AF700); CD45RA (HI100: PE-Cy7); CCR7 (G043H7: AF700); CD103 (Ber-ACT8: PE-Cy7); CD69 (FN50: Bv605); Interferon-γ (45.B3: Bv421, AF488); Perforin (BB-D48: PE); CD107a (H4A3: Bv421); Interleukin-2 (MQ1-17H12: Bv711); and TNF-α (MAb11: Bv650) all from Biolegend; CD4 (RPA-T4: APC-Cy7) and Granzyme B (GB11: PECF594) from BD Bioscience; and Eomesodermin (WD1928: eFluor660) from eBioscience.

To identify HIV-specific CD8^+^ T cell responses, DMNCs and PBMCs were stimulated using PMA/Ionomycin (Sigma Aldrich) as a positive control, while a combination of Gag-, Nef-, and Pol-peptide pools (all peptides, JPT Innovative Peptide Solutions) were used as test stimulants. Briefly, cells were incubated at a concentration of 1 × 10^6^ cells in 200 μL of complete medium (RPMI 1640 plus l-glutamine, Life Technologies), 1% HEPES buffer, 1% penicillin/streptomycin, and 10% FBS (all Sigma-Aldrich) (200 μL per condition) in the presence of stimulant (or unstimulated control), anti-CD28, anti-CD49d, BD GolgiPlug, and BD GolgiStop (all BD Biosciences) for a total of 6 hours at 37°C in a 5% CO_2_ incubator. After stimulation, cells were harvested and washed in PBS. Cells were labeled with the amine reactive dye LIVE/DEAD Fixable Aqua (Molecular Probes, Invitrogen) prior to incubation with antibodies against surface proteins. Staining for cytokines, transcription factors, and cytolytic granules was done after fixation/permeabilization with BD Cytofix/Cytoperm (BD Biosciences). The cells were then acquired on a flow cytometer. For all flow cytometric assays, we used an LSRFortessa and FACS Aria equipped with FACSDIVA software (BD Biosciences), at least 50,000 events in the CD8^+^ T cell gate were acquired. We analyzed the data using FlowJo software (version 10.7.1, Tree Star), PESTLE/SPICE (NIH).

### Viral load measurements.

Duodenal tissue supernatant was obtained by concentrating 15 mL of duodenal tissue collection media using Amicon Ultra Centrifugal filters (Millipore) returning a volume of 2 mL. Plasma was collected by centrifuging whole blood to separate the plasma and red blood cells. HIV RNA levels in the duodenal lining fluid and plasma were measured by quantitative PCR. In brief, 1 mL of specimen was loaded into HIV-1 viral load assay cartridges with integrated reaction tubes (Xpert HIV-1 Viral Load, Cepheid). The cartridges were loaded into a GeneXpert instrument system which carried out RT-PCR and viral load was quantified by the instrument software. Detection level as defined by the manufacturer was 40 copies/mL.

### Statistics.

Net HIV-specific responses were calculated by subtracting background signals in the unstimulated control from antigen specific responses in the stimulated condition (29, 51). The threshold for a positive response was set at 0.05% of CD8^+^ T cells (51, 52), and values below this threshold were assigned as zero. We performed statistical analyses and graphical presentation using GraphPad Prism 9 (GraphPad Software). Statistically significant differences between groups were determined using parametric and nonparametric tests, Mann–Whitney test (for 2 unpaired groups), the Wilcoxon’s matched-pairs signed rank test (for paired samples), Kruskal–Wallis test (for 3 or more groups of unpaired samples), or repeated measures of 1-way ANOVA test (for 3 or more groups of paired samples), respectively. Post hoc corrections were applied. A 2-stage step-up method of Benjamini, Krieger, and Yekutieli or the Holm–Šídák test was used to correct for multiple comparisons. All error bars indicate IQR with median. The association between parameters was measured using Spearman’s test. A *P* value less than 0.05 was considered significant.

### Study approval.

All enrolled participants gave written informed consent as per the protocol approved by the Kamuzu University of Health Sciences Research Ethics Committee (COMREC protocol P.09/17/2284).

## Author contributions

ZMN and KCJ conceived the idea. KCJ, TN, MAG, and ZMN designed the study. LM, TK, AL, NJ, and IK carried out the laboratory experiments and analyses. PP and AK coordinated subject recruitment and obtained all clinical samples. LM, NJ, IK, and AL processed the samples. KCJ, LM, and ZMN wrote the manuscript. All authors approved the final manuscript.

## Supplementary Material

Supplemental data

## Figures and Tables

**Table 1:**
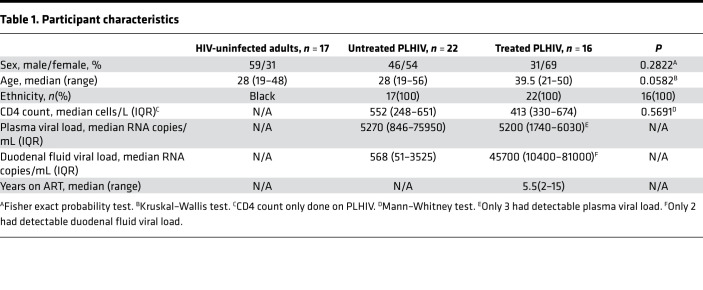
Participant characteristics

## Version 1 02/08/2022

Electronic publication
