# Supplementary material for: Differential localization and limited cytotoxic potential of duodenal CD8+ T cells
Source: JCI Insight. 2022 Feb 8;7(3):e154195. doi: 10.1172/jci.insight.154195 (PMC8855799; doi:10.1172/jci.insight.154195)
Supplement: Supplemental data [file jciinsight-7-154195-s289.pdf]

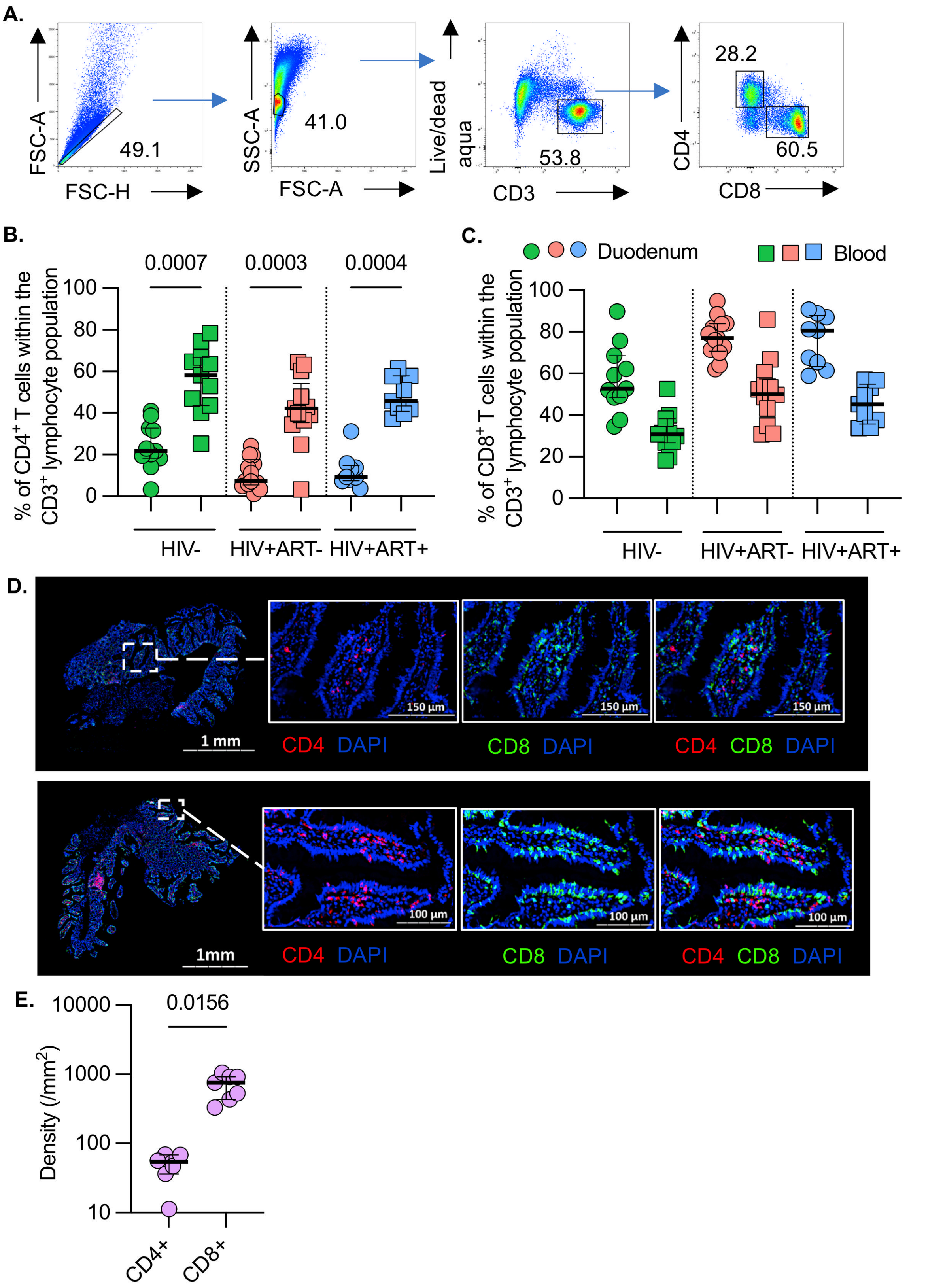

Supplementary Figure 1. Flow cytometric and immunohistochemistry analysis of CD4⁺ and CD8⁺ T cells (**A**) Representative flow cytometry gating strategy for characterising CD4⁺ and CD8⁺ T cells in DMNCs from an HIV-uninfected control**.** DMNCs were stained with

fluorochrome-conjugated antibodies against surface markers of interest. The flow cytometry dot plots were obtained by gating on singlets, lymphocytes, live/dead aqua- CD3+ cells, then CD8+ cells or CD4+ cells (**B**) Comparison of the frequency of $CD4^+$ T cells in the duodenal mucosa and peripheral blood from ART-untreated and ART-treated PLHIV. (**C**) Comparison of the frequency of $CD8^+$ T cells in the duodenal mucosa and peripheral blood from ART-untreated and ART-treated PLHIV. Data were analyzed using Kruskal-Wallis test and adjusted for multiple comparisons (Two-stage Benjamini, Krieger, & Yekutieli) for different participant groups. (**D**) Immunohistochemistry images of duodenal biopsy section showing CD4 (red), CD8 (green) and DAPI (blue). (**E**) Density of $CD4^+$ and $CD8^+$ T cells within duodenal tissue from PLHIV (ART-, n=2; ART+, n=2) and HIV-uninfected (n=3) individuals. Data were analyzed using Mann-Whitney test.

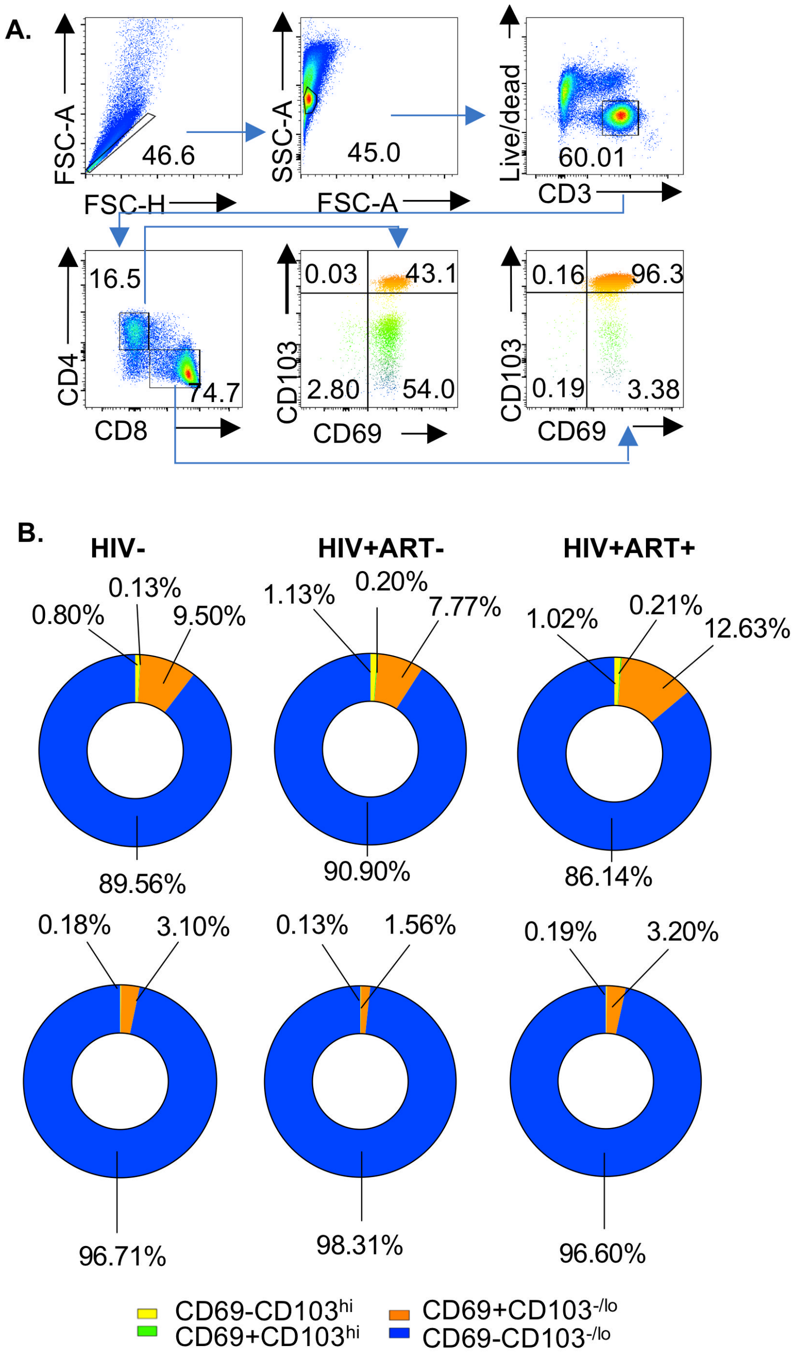

Supplementary Figure 2. Characterization of CD69 and CD103 expressing CD4^{+} and CD8^{+} T cell populations. (**A**) Representative flow cytometry gating strategy to identify duodenal CD4^{+} and CD8^{+} T cell populations expressing different patterns of CD69 and CD103. DMNCs were stained with fluorochrome-conjugated antibodies against surface markers of interest. The flow cytometry dot plots were obtained by gating on singlets, lymphocytes, live/dead aqua- CD3+ cells, CD8+ cells or CD4+ cells, then CD69 vs CD103 (**B**) Pie charts representing the median proportion of peripheral CD8^{+} and CD4^{+} T cells expressing different combinations of CD69 and CD103 in different participant groups (HIV−, n = 13; ART-, n = 13; ART+, n=10).

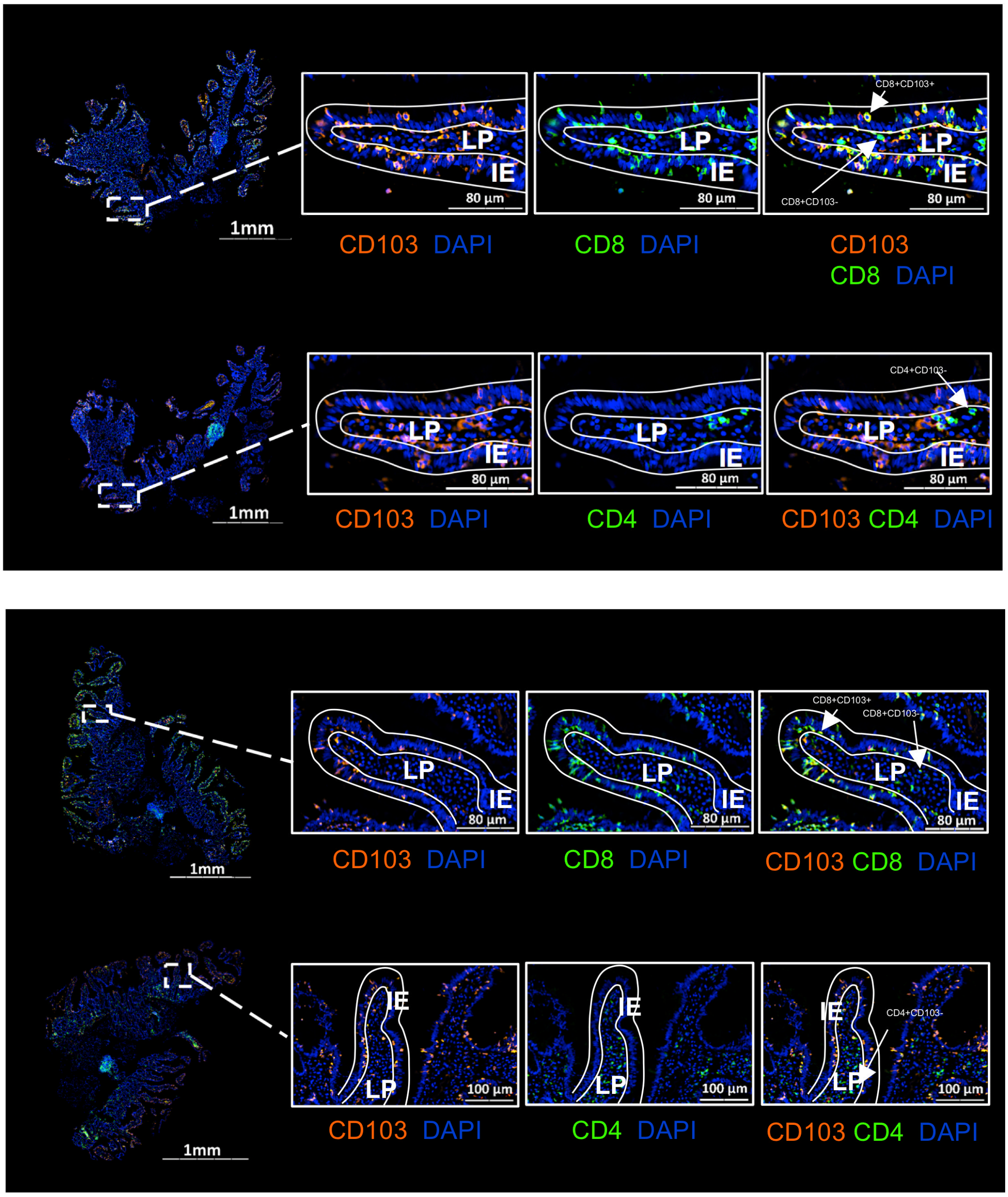

Supplementary Figure 3. Immunohistochemistry analysis of the spatial localisation of duodenal CD4$^+$ and CD8$^+$ T cells. Representative Immunohistochemistry images of duodenal biopsy sections showing CD4 (green), CD103 (orange) and DAPI (blue) staining within the delineated compartments defined as the intraepithelial (IE) region and the lamina propria (LP)

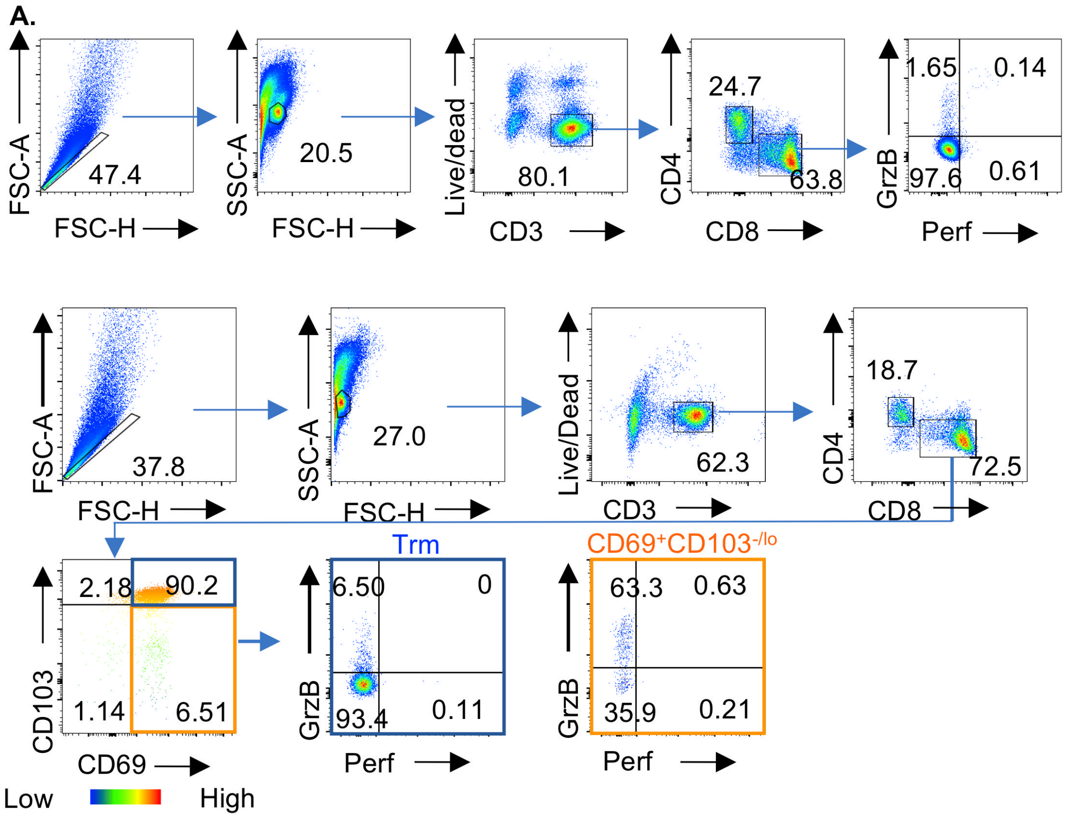

Supplementary Figure 4. Representative flow cytometry gating strategies for identifying pre-formed perforin and granzyme B co-expression by duodenal CD8$^+$ T cells. (**A**) Flow cytometry gating strategy to identify bulk duodenal CD8$^+$ T cell populations expressing different patterns of perforin and granzyme B. DMNCs were stained with fluorochrome-conjugated antibodies against surface markers of interest. The flow cytometry dot plots were obtained by gating on singlets, lymphocytes, live/dead aqua- CD3+ cells, CD8- cells, then perforin vs granzyme B (**B**) Representative flow cytometry gating strategy to identify intraepithelial (IE) and lamina propria (LP) CD8$^+$ T cell populations expressing different patterns of perforin and granzyme B. The flow cytometry dot plots were obtained by gating on singlets, lymphocytes, live/dead aqua- CD3+ cells, CD8- cells, then CD69+CD103hi and CD69+CD103-/lo, followed by perforin vs granzyme B

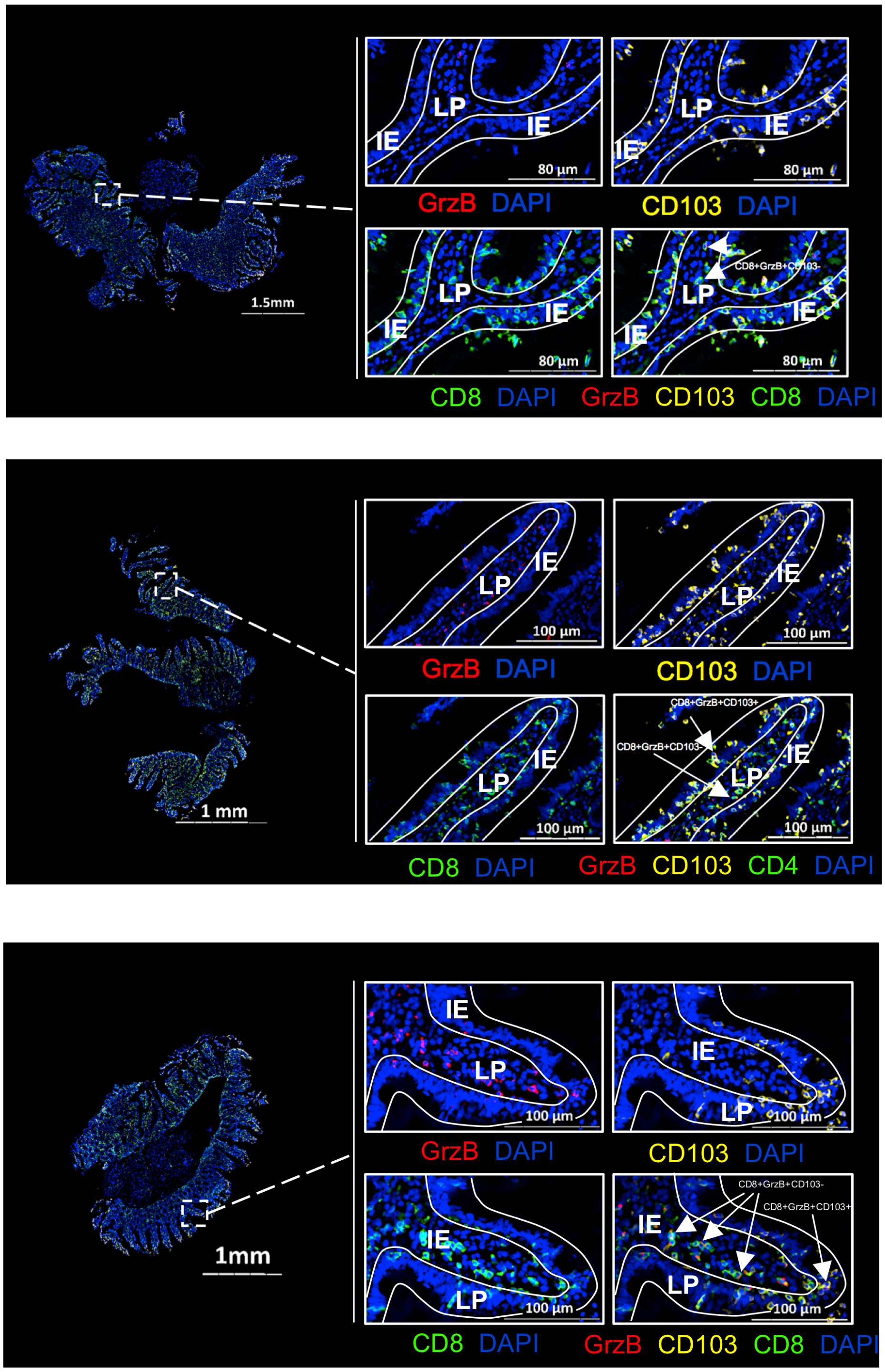

Supplementary Figure 5. Immunohistochemistry analysis of granzyme B expression by duodenal CD8$^+$ T cells. Representative immunohistochemistry images of duodenal biopsy section showing granzyme B (red), CD103 (yellow), CD8 (green), and DAPI (blue) staining.

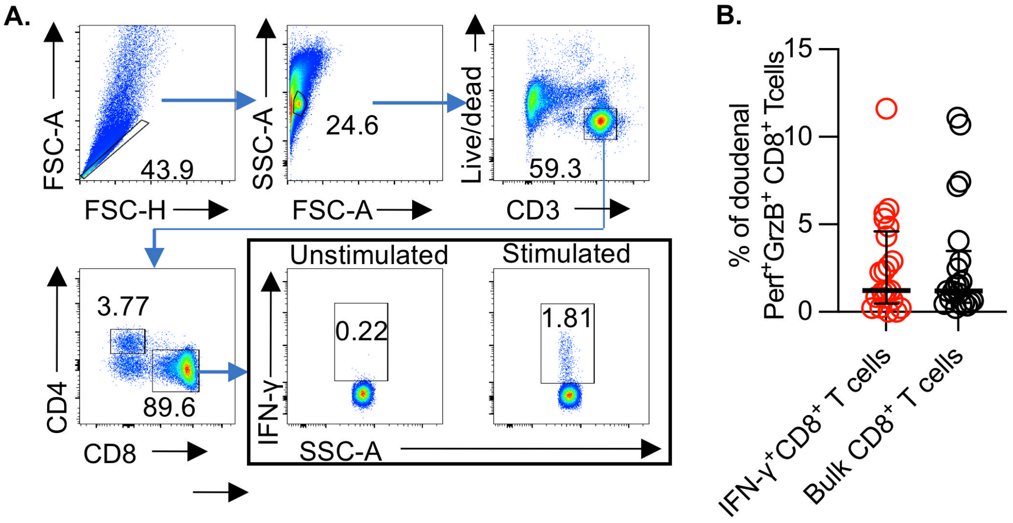

Supplementary Figure 6. Detection of HIV-specific CD8^{+} T cells. (**A**) Representative flow cytometry plot showing detection of duodenal IFNγ producing CD8^{+} T cells. DMNCs from PLHIV were stimulated with pooled HIV Gag, Pol and Nef peptides for 6 hours. The stimulated cells were stained with fluorochrome-conjugated antibodies against surface markers of interest. The flow cytometry dot plots were obtained by gating on singlets, lymphocytes, live/dead aqua- CD3+ cells, CD8- cells, then IFNγ+ cells. (**B**) Comparison of the frequency of perforin and granzyme B co-expressing HIV-specific and bulk duodenal CD8^{+} T cells (ART-, n=13; ART+, n=7). Data were analyzed using Wilcoxon test.
